# Signalling mechanisms in the cardiovascular protective effects of estrogen: With a focus on rapid/membrane signalling.

**DOI:** 10.1016/j.crphys.2021.03.003

**Published:** 2021-03-28

**Authors:** Ana-Roberta Niță, Greg A. Knock, Richard J. Heads

**Affiliations:** a School of Bioscience Education, Faculty of Life Sciences and Medicine, King’s College London, UK; b School of Immunology and Microbial Sciences, Faculty of Life Sciences and Medicine, King’s College London, London, UK; c Cardiovascular Research Section, King’s BHF Centre of Research Excellence, School of Cardiovascular Medicine and Sciences, Faculty of Life Sciences and Medicine, King’s College London, UK

**Keywords:** Estrogen receptor, Cardiovascular, ER, GPER, Rapid signaling, Endothelial, Vascular smooth muscle

## Abstract

In modern society, cardiovascular disease remains the biggest single threat to life, being responsible for approximately one third of worldwide deaths. Male prevalence is significantly higher than that of women until after menopause, when the prevalence of CVD increases in females until it eventually exceeds that of men. Because of the coincidence of CVD prevalence increasing after menopause, the role of estrogen in the cardiovascular system has been intensively researched during the past two decades *in vitro*, *in vivo* and in observational studies. Most of these studies suggested that endogenous estrogen confers cardiovascular protective and anti-inflammatory effects. However, clinical studies of the cardioprotective effects of hormone replacement therapies (HRT) not only failed to produce proof of protective effects, but also revealed the potential harm estrogen could cause. The “critical window of hormone therapy” hypothesis affirms that the moment of its administration is essential for positive treatment outcomes, pre-menopause (3–5 years before menopause) and immediately post menopause being thought to be the most appropriate time for intervention. Since many of the cardioprotective effects of estrogen signaling are mediated by effects on the vasculature, this review aims to discuss the effects of estrogen on vascular smooth muscle cells (VSMCs) and endothelial cells (ECs) with a focus on the role of estrogen receptors (ERα, ERβ and GPER) in triggering the more recently discovered rapid, or membrane delimited (non-genomic), signaling cascades that are vital for regulating vascular tone, preventing hypertension and other cardiovascular diseases.

## Highlights

• Effects of estrogen on vascular smooth muscle cells (VSMCs) and endothelial cells (ECs).
• Focus on the role of estrogen receptors (ERα, ERβ and GPER) in rapid or membrane delimited (non-genomic) signaling.
• Role regulating the vascular tone, preventing hypertension and other cardiovascular diseases.

## Graphical abstract

**Figure:**
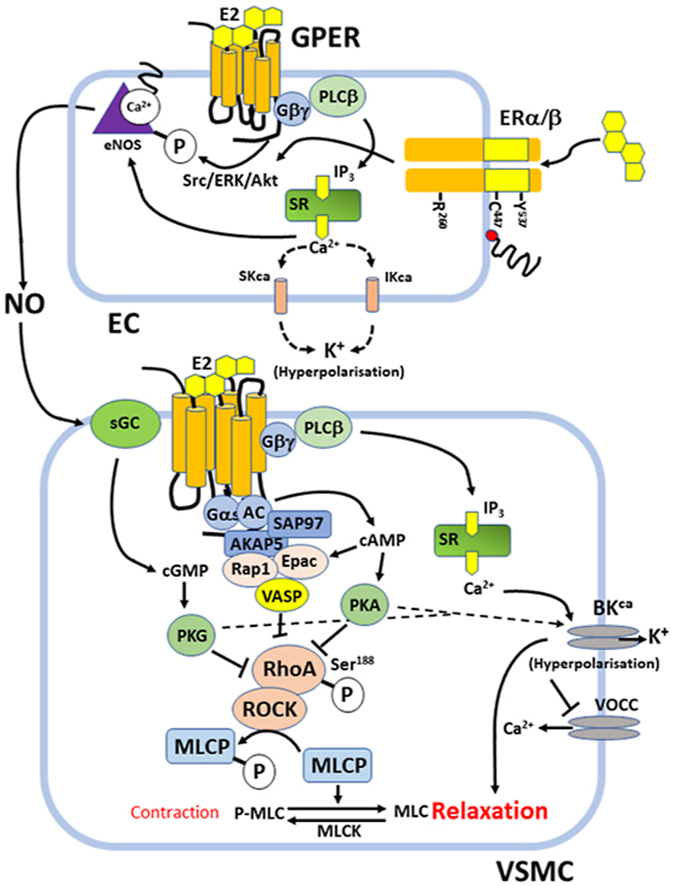

## Abbreviations

CVD: Cardiovascular disease
HRT: hormone replacement therapy
SMCs: smooth muscle cells
RAAS: renin angiotensin aldosterone system
Akt: PKB (protein kinase B)
PI3K: Phosphoinositide 3-kinase
HSP90: heat shock protein 90
c-Src: proto-oncogene tyrosine-protein kinase Src
ERK: Extracellular signal regulated kinase
MAPK1: Mitogen-activated protein kinase 1
NO: nitric oxide;
iNOS: inducible nitric oxide synthase
eNOS: endothelial nitric oxide synthase
CAM: Calmodulin
HUVEC: human umbilical vein endothelial cells
UAECs: uterine artery endothelial cells
ECs: endothelial cells
VSMCs: vascular smooth muscle cells
CM: Cardiomyocyte
E2BSA: estradiol conjugated to bovine serum albumin
Grb2: Growth factor receptor-bound protein 2
GPCR: G protein coupled receptor
Cos7: fibroblast-like cell line;
EGFR: epidermal growth factor receptor
MLCK: myosin light chain kinase
MLCP: myosin light chain phosphatase
IP3: inositol trisphosphate
DG: diacylglycerol
PIP2: phosphatidyl inositol 4,5 bisphosphate
SD: Sprague Dawley rats
ET-1: endothelin 1
VDCC: voltage dependent calcium channel
MAGUK: membrane associated guanylate kinase
PCAs: porcine coronary arteries
PCASMCs: porcine coronary artery smooth muscle cells
ARB: angiotensin receptor blocker
ROS: reactive oxygen species
L-NAME: L-N^G^-nitroarginine methyl ester
PVAT: perivascular adipose tissue
PVRFs: PVAT-derived relaxing factors
sGC: soluble guanylate cyclase
PKG: protein kinase G
cGMP: cyclic guanosine monophosphate
cAMP: cyclic adenosine monophosphate
MYPT1: myosin phosphatase target subunit 1
MLCK: myosin light chain kinase
MLCP: myosin light chain phosphatase
CcOX: cytochrome C oxidase
EAhy926 ​cells: human endothelial-like cells
PRMT1: protein arginine methyl transferase 1
NOX4: NADPH Oxidase 4
Kv: voltage-dependent K^+^ channels
BKca: large conductance calcium- and voltage-dependent K^+^ channels
VASP: vasodilator stimulated phosphoprotein
LVEDP: left ventricular end diastolic pressure
PGE_2_: prostanglandin E_2_
IL-6: interleukin-6
IL-1β: interleukin-1 beta
TNFα: tumour necrosis factor alpha
NFκB: nuclear factor kappa B
CXCL8: CX chemokine ligand 8
COX-2: cyclooxygenase-2 (Ptgs2)
TLR4: Toll-like receptor-4
TAC: transaortic constriction

## 1 Introduction

Estrogens are a class of steroid hormones that are mainly synthesized by the ovaries, adrenals and by the placenta in pregnancy. The main estrogens are 17β-estradiol (E2) (the most potent and main circulating one); estrone (E1) and estriol (E3). There are a number of extragonadal sites where low quantities of E2 are produced. These remain the only endogenous estrogen source in postmenopausal and ovariectomized women and in men. These sites include the vascular endothelium, aortic SMCs, adipose, brain, and bone tissues (see for instance (Barakat et al., 2016)). Extragonadal E2 acts as a paracrine or autocrine modulator in its origin tissue, so for instance, would have a paracrine action in the vasculature (Simpson, 2003). Besides their role in developing the primary and secondary sexual characteristics of women, multiple studies have shown that they can also protect the cardiovascular system of pre-menopausal women and men against disease (see (Novella et al., 2019; Iorga et al., 2017)). In males, conversion of testosterone to E2 by aromatase may be particularly important, emphasised by the fact that cardiac aromatase activity in male mice was decreased following transaortic constriction (TAC)-induced left ventricular heart failure and which was normalized by E2 treatment (Iorga et al., 2016) (and see (Iorga et al., 2017)). Menopause is defined as the time at twelve months after the last menstrual cycle (period) in women. The menopausal transition can last up to seven years and usually begins between the ages of 45 and 55 years old. It also correlates with decreasing levels of ovarian hormones and can be triggered by surgical oophorectomy (removal of the ovaries).

In the search for the mechanisms by which estrogens exert their genomic and rapid, non-genomic actions in the vasculature, three main types of estrogen receptors (ER) were found to exist in both vascular smooth muscle cells (VSMC) and endothelial cells (EC): ERα, ERβ and GPER1 (G-protein coupled estrogen receptor 1; formerly GPR30: G-protein coupled receptor 30), (see also (Levin, 2009; Murphy, 2011; Menazza and Murphy, 2016; Zimmerman et al., 2016)). By binding these receptors, estrogen can induce vasodilation, reduce inflammation, act as a potent antioxidant, and alter gene expression. Furthermore, estrogens are well-known for their actions in reducing plasma cholesterol levels and its deposition in the wall of arteries (Austin, 2000).

**Fig. 1:**
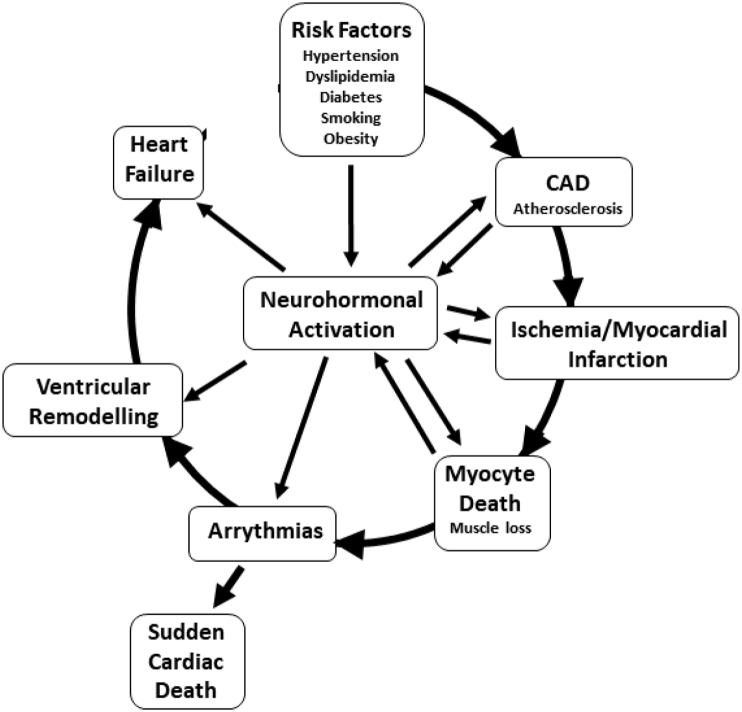
The cardiovascular disease continuum.

By inducing vascular relaxation, E2 is thought to be able to mitigate against hypertension (see (Ashraf and Vongpatanasin, 2006; Rafikova and Sullivan, 2014). However, it is a complicated picture because contrary to this is the possibility that long-term exposure to estrogen in the form of oral contraceptive or HRT medications may increase hypertension, due to the buildup of superoxide radicals (Subramanian et al., 2011; MohanKumar et al., 2011). However, this may be more prevalent with synthetic estrogens rather than natural estradiol (Dubey et al., 2002). Hypertension is an elevation of systolic and/or diastolic blood pressure. It is a major risk factor in the initiation and progression of cardiovascular disease ie the “cardiovascular disease continuum” (Dzau et al., 2006), (as shown in Fig. 1), and is also associated with renin-angiotensin-aldosterone system (RAAS) activation. Hypertension can be aggravated (or initiated) by other risk factors such as obesity, smoking, male sex, lack of physical activity, stress, old age, excess salt in the diet and by estrogen loss due to menopause. High pressure damages the endothelium of the arteries thus promoting the development of atherosclerosis (Nakanishi et al., 2017). Furthermore, it increases ventricular afterload (the force the left ventricle has to generate to expel blood into the aorta), leading to cardiac hypertrophy and demand ischemia (Murphy et al., 2009). Furthermore, female sex is associated with reduced tolerance against acute ischemic events and increased risk of demand ischemia (Podesser et al., 2007). Low resistance, high flow vascular systems such as the brain and kidney, are the tissues that are most affected by hypertension. Increased pressure in a low resistance microcirculation, exposes it to even higher pressures and pressure pulsatility compared to other organs. This damages the microcirculation and can lead to end organ damage, renal failure and stroke. Therefore, interrupting this vicious cycle, can substantially decrease the morbidity and mortality associated with it.

The effects of estrogen in reducing hypertension and other risk factors have been researched *in vivo*, *in vitro* and in observational studies. Overall, experimental research has suggested that estrogen is protective of both the heart and the vasculature. On the other hand, comprehensive clinical research on hormone replacement therapy (HRT) in post-menopausal women has failed to provide strong evidence for protection and even revealed the potential harm it can cause to the patients. The “theory of timing and opportunity” explained that whether estrogen administration is beneficial or deleterious, depends greatly on the stage of menopause (Menazza and Murphy, 2016; Dehaini et al., 2018).

## 2 Estrogen receptors

Estrogen signaling in target organs depends on estrogen receptors (ERα, ERβ and GPER). These receptors can either work synergistically or antagonistically, but they exert similar actions in ECs and VSMCs. By activating these receptors, E2 can trigger both genomic and non-genomic actions (Menazza and Murphy, 2016; Acconcia et al., 2005). Although the role of these receptors has been extensively studied in arteries, less is known regarding their role in the venous system.

### 2.1 Estrogen receptor (ER) alpha (α) and beta (β)

The gene for human estrogen receptor alpha (ERα) is encoded on chromosome 6. It has 595 amino acids that are arranged in 5 different domains and its molecular size is 66 ​kDa. ERα is found both in association with the cell membrane, allowing fast, ‘non-genomic’ estrogen signaling and in the nucleus, mediating estrogen’s longer term, ‘genomic’ actions (Levin, 2009; Murphy, 2011; Acconcia et al., 2005). However, as discussed below, the more rapid signaling from the plasma membrane does impinge on changes in gene transcriptional regulation, via intermediate kinase mediated signaling and via co-operation with nuclear ER, and therefore ‘non-genomic’ signaling may be more accurately referred to as ‘membrane delimited’ signaling (see (Menazza and Murphy, 2016)). Also, other intracellular localisations of ER have been described, such as mitochondria, endoplasmic reticulum and Golgi (Hammes and Levin, 2007) (Razandi et al., 2013). Localization of ERα in endothelial cells (ECs) and VSMCs has been extensively investigated (see (Acconcia et al., 2005; Gourdy et al., 2018).

**Fig. 2:**
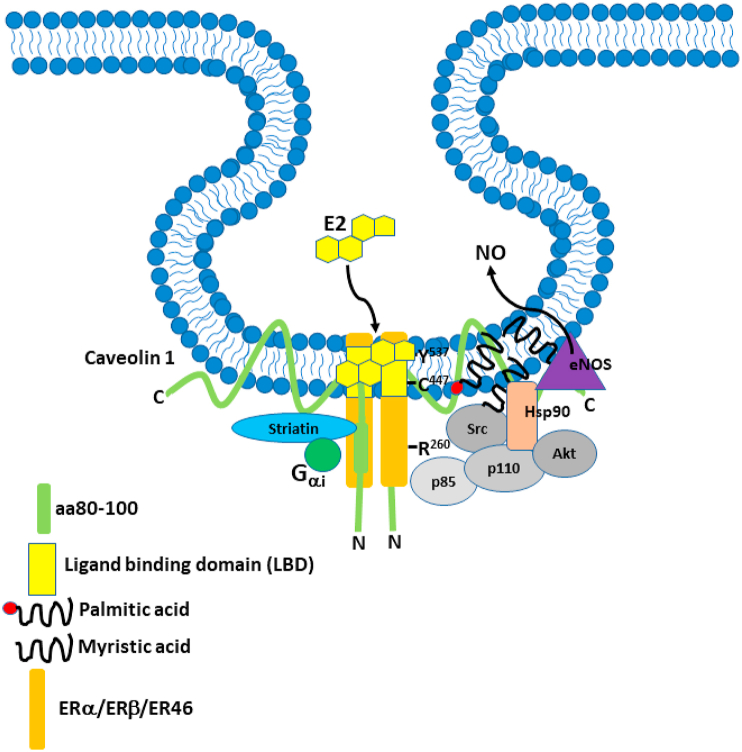
The Estrogen Receptor Complex. ERα, ERβ or ER46 is complexed with Gαi, c-Src TK, PI3K, Akt, Hsp90 and eNOS in caveolae, where it assembled on caveolin 1 with striatin. In this depiction ER is shown assembled on caveolin 1 and spanning the membrane. There is evidence that ER presents an ectodomain, although it is not clear whether the C-terminal ligand binding domain is extracellular or in the membrane, because E2 is lipid soluble and can cross the membrane. ER assembles on the aa80-100 region of the caveolin 1 ​N-terminus. ER is palmitylated on Cys^447^ which anchors it to the membrane. The recruitment of Src TK and PI3K can occur on the methylated Arg^260^. However, phosphorylation of Tyr^537^ has also been reported to recruit Src TK via its SH2 group, although this would probably occur in a model where ER is tethered to the inside of the membrane rather than spanning the membrane. C-Src tyrosine kinase is also myristylated on Gly^2^. In endothelial cells estrogen (E2) binding to this complex rapidly initiates a signaling cascade leading to the activation of eNOS and NO production. P85 and p110 are regulatory subunits of PI3K. Rapid signaling is essential for inhibiting VSMC proliferation, inducing vasorelaxation and endothelial cell proliferation and migration. Adapted from various sources, including (Kim and Bender, 2005).

ERα does not contain a typical trans-membrane domain, but palmitoylation (covalent attachment of palmitic acid to an amino acid residue) at Cys^477^ regulates membrane trafficking and localization to the plasma membrane, where it is concentrated in caveolae, associated with caveolin-1 (Acconcia et al., 2005; Chambliss et al., 2002; Levin, 2014). Cys^477^ is contained within a 9 aa palmitoylation motif which is critical for full palmitoylation of ERα. In addition, methylation of Arg^260^ in the DNA binding domain by Protein Arginine Methyl Transferase 1 (PRMT1), plays a role in exclusion of ER from the nucleus and cytoplasmic localization and trafficking. Furthermore, in breast cancer cells Arg^260^ methylation triggers interaction with the p85 subunit of phosphatidyl inositol-3-kinase (PI3K), c-Src tyrosine kinase (c-Src) and recruitment of Focal Adhesion Kinase (FAK) (Le Romancer et al., 2008). Approximately 5–10% is found in the PM but it is also found in the endoplasmic reticulum and mitochondria (see (Menazza and Murphy, 2016)). It has also been shown that in ECs ERα exists as a 46 ​kDa N-terminal truncated splice variant, ER46, that also complexes with caveolin-1, Akt, HSP90, PI3K, c-Src and endothelial Nitric Oxide Synthase (eNOS) in caveolae (see Fig. 2) (Kiss et al., 2005). Li et al. showed that ER46 is more efficient at generating Nitric Oxide (NO) from eNOS than the full length ER66, but that ER66 is more efficient at triggering genomic ER signaling. Also, interestingly, antibody accessibility of the C-terminus of ER46 in intact cells suggests that it may at least partially span the caveolar PM. Furthermore, ER46 is activated efficiently by membrane impermeant E2, therefore the ligand binding domain is extracellular (Li et al., 2003).

Estrogen Receptor beta (ERβ) in humans is a 530-amino acid protein that has a molecular size of 54 ​kDa and its gene is encoded on chromosome 14. Just like ERα it has 5 domains all having distinct functions. Fewer investigations were made on ERβ because it was not cloned until 1996 (Acconcia et al., 2005). Also, the relative amounts of ERα and ERβ vary depending on the cell type. In an early study of ERα and ERβ mRNA distribution, it was shown that ERα mRNA was expressed in all tissues and ERβ mRNA mainly in reproductive tract except for mammary and testes. Hypothalamus and lung were high for ERα and ERβ, but ERβ was downregulated in ERα knockouts, suggesting that ERβ expression was dependent on ERα (Couse et al., 1997). ERs are expressed in both ECs and VSMCs, and E2 affects blood vessel structure as well as function (Kim et al., 2008). ERα knockout mice express a vascular as well as metabolic phenotype (Luksha and Kublickiene, 2009). The differences between ERα and ERβ expression are complex and appear to depend on the vascular bed as well as sex and disease state. Ma et al. (2010) compared the relaxation responses of male and female rat aortic VSMCs to ERα, ERβ and GPER agonists against the constrictors AngII, PE and KCl and examined the intracellular distribution of the different receptors as well as mRNA and protein levels. AngII and PE caused less contraction in female VSMCs, although in the absence of agonist, this may be due to genomic effects unless the cells themselves produce intrinsic E2 which acts in an autocrine fashion. The ERα agonist PPT caused similar relaxation to E2, suggesting a predominance of the ERα mediated effect. The ERβ agonist DPN caused mainly relaxation to PE and KCl, suggesting an ERβ-Ca^2+^ channel interaction. The ERα/ERβ antagonist ICI 182, 780 (which is a GPER partial agonist) did not reduce VSMC contraction, suggesting little role for GPER, although they didn’t directly test a GPER agonist (ie G-1). ERα and ERβ expression was higher in females, in keeping with the smaller contraction in female VSMCs and were largely expressed in the nucleus. Given that this was an analysis of the acute effects on VSMC contraction, there may have been sensitivity issues with the immune detection in other compartments. GPER expression was similar in males and females, and mainly non-nuclear in localization. However, although PPT increased the nuclear localization of ERα, DPN had no effect on ERβ localization and ICI did not increase GPER localization to the cell surface. These results suggest a predominance of ERα mediated responses in VSMCs, at least from the aorta. However, VSMC contraction/relaxation *in vitro* may not be fully representative of the intact artery.

ERα and ERβ are highly homologous and have approximately 56% homology in the ligand binding domain and 95% in the DNA binding domain (Mosselman et al., 1996). ERβ distribution and role varies within different vessels with different cell types. For example, in embryonic ovine intra-pulmonary arteries it is mainly associated with non-caveolar sites of the plasma membrane of the endothelial cells and it was found to be the main eNOS activator compared to ERα. On the other hand, ERα is the predominant activator of eNOS in adult models (Chambliss et al., 2000), but again may depend on the vascular bed, as it was similar in UAECs (Pastore et al., 2016). Similar to ERα, ERβ can also activate PI3K, Akt, and the Extracellular Signal Regulated Kinase 1 and 2 (ERK1/2) pathways (Wu et al., 2011). In the proximity of caveolae ERα and ERβ signaling are essentially the same ie leading to activation of Gα and Gβγ, increases in Ca^2+^ and cAMP and activation of c-Src, PI3K and the distal kinases Akt and ERKs (see for instance (Sugiyama et al., 2010)). As mentioned above, in VSMCs, ERβ is primarily found in the nucleus but can also be found in lower quantities at the plasma membrane, associated with caveolin-1 (Chambliss et al., 2002).

### 2.2 G-protein coupled estrogen receptor (GPER1/GPR30/GPER)

GPER1/GPR30 was discovered as an orphan G protein coupled receptor with 7 transmembrane helices (Pietras and Szego, 1977; Barton et al., 2018). Its gene is located on chromosome 7p22.3 in humans and 5g2 in mouse, a region that is implicated in arterial hypertension and familial hyperaldosteronism (FH). However, the role of GPER in hypertension is complex (see for instance (Zimmerman et al., 2016; Haas et al., 2009; Prossnitz and Barton, 2011; Meyer et al., 2009)). FH is associated with several genes including AT1, CYP11b2, MEN1, PKRAR1B, RBaK, PMS2 and GNA12 on the 7p22 locus (Stowasser and Gordon, 2007), and a later study identified a comprehensive gene list associated with FHII on the 7p22 locus, but the precise mutations remain elusive (Carss et al., 2011). Furthermore, this did not include polymorphisms in the GPER gene. However, this does not preclude a possible role for GPER loss-of-function in other forms of primary hypertension. Recently an association was found between lower GPER levels and hypertension in post-menopausal but not pre-menopausal women (Liu et al., 2018). However, the complexity of the role of GPER appears to be due in part to the fact that it is disputed whether or not GPER also functions as an aldosterone receptor (Gros et al., 2013; Briet and Schiffrin, 2013; Waghulde et al., 2019). Nevertheless, female wild type mice are protected against AngII-induced hypertension whereas as GPER knockout mice are not. Furthermore, this was associated with increased oxidative stress as evidenced by increased reactive oxygen species (ROS), NADP/NADPH and NADPH Oxidase 4 (NOX4). The GPER agonist G-1, the angiotensin receptor blocker (ARB) losartan and NOX4 siRNA all decreased NOX4 mRNA and protein and blocked the effects of AngII. The G-1 protective effects were also blocked by adenylate cyclase inhibition and mimicked by phosphodiesterase inhibition, highlighting the importance of the cAMP pathway downstream of GPER in the protective effects (Ogola et al.G, 2019). The involvement of NOX4 is a surprising finding given that the main generator of vascular ROS (ie, superoxide, O_2_^−^) in response to AngII is NOX1/2 rather than NOX4, and NOX4 is regarded as protective as it generates H_2_O_2_ rather than superoxide which is pro-relaxant, probably by activating eNOS, SR CICR and BKca channel activation (Zhang et al., 2018; Knock, 2019). GPER has also been reported to protect against high salt-induced hypertension in ovariectomized rats (Gohar et al., 2020).

Structurally, GPER is not related to any of the classical estrogen receptors. E2 affinity for ERα and β is ten times higher than that for GPER. However, cells that express both GPER and ERα such as coronary vessel cells (either VSMCs or ECs), may respond to coordinated signaling of the two receptors (Prossnitz and Barton, 2014). Evidence for functional crosstalk between ERα and GPER was obtained in porcine coronary artery, where acute NO-dependent vasodilation was observed in response to ERα selective agonism, but blocked when E2 activated all three receptors simultaneously. However, these observations are complicated by the fact that ERα-selective agonists such as PPT may also activate GPER (Traupe et al., 2007; Petrie et al., 2013).

When a GPER1 specific agonist was administered intracellularly in VSMCs of arterial vessels, the vasodilatory response was faster compared to that resulted from external administration. Also the use of differentially permeable E2 derivatives have suggested that membrane permeability is a necessity for rapid signaling by GPER, suggesting that the intracellular GPER is the main functional pool (Revankar et al., 2007). This agrees with the fact that most GPER is localized intracellularly. Studies using fluorescent GFP-tagged GPER appeared to confirm this as high levels were found in the endoplasmic reticulum and Golgi, but not the PM (Revankar et al., 2005). However, although it is now generally accepted that GPER1 is predominantly located intracellularly in vascular cells (Haas et al., 2009; Prossnitz et al., 2008), it is not clear whether, or to what extent, intracellular signaling contributes to its function. Estrogen binding to GPER induces calcium mobilization possibly via EGFR transactivation (Filardo et al., 2000), rather than via IP_3_-mediated action, so the relative contributions of the two mechanisms and the localization of the effect are debated (Menazza and Murphy, 2016). Furthermore, PLCγ docks with EGFR and is activated by tyrosine phosphorylation (Lappano et al., 2013), which may link the two effects. Also, GPER appears to be constitutively endocytosed and to have a high rate of turnover and therefore this may explain the apparent lack of PM located GPER and its accumulation in endosomes (Cheng et al., 2011). This may be important in view of the growing recognition of endosome signaling in its own right, so it is possible that GPER signals from this compartment rather than simply transits through it. Therefore, the high levels in the endoplasmic reticulum and Golgi may simply reflect high rates of synthesis and trafficking through these compartments during protein synthesis and post-translational processing (Menazza and Murphy, 2016). Thus, evidence for a direct intracellular signaling role of GPER remains inconclusive. However, there is evidence for rapid G-protein-dependent signaling in ERα and ERβ negative or inhibited cells, suggesting a direct role for GPER which is independent of ERα an or ERβ (Ullrich et al., 2008) (and see (Barton et al., 2018)).

The actions of GPER activation varies with each type of vascular bed. For example, GPER selective activation in mesenteric arteries leads to endothelium-dependent vasorelaxation contributed to by signaling cascades activated in both VSMCs and ECs (Haas et al., 2009). Likewise, porcine coronary artery relaxation induced by the GPER selective agonist G-1 is endothelium-dependent via eNOS activation (Meyer et al., 2010). However, other studies suggest that vasorelaxation in aorta and coronary arteries in response to G-1 can occur in an endothelium-independent manner (Lindsey et al., 2009; Yu et al., 2011; Jiang et al., 1991). In these studies, eNOS inhibition by L-N^G^-nitroarginine methyl ester (L-NAME) did not affect the vasodilatory response. Furthermore, Yu et al. (2011) suggest that the endothelium independent effect of G-1 in coronary VSMCs is mediated via a large conductance Ca^2+^-activated potassium (BK) channel (see also (Menazza and Murphy, 2016; Zimmerman et al., 2016)). Therefore, there are endothelium-dependent and -independent effects of GPER signaling.

Extensive analysis of GPER knockout mice show relatively subtle phenotypes, compared to the extensive and reproducible differences seen in ERα knockout mice (see (Prossnitz and Hathaway, 2015)). For instance, an age-dependent effect on blood pressure was observed in female mice, since an increase in blood pressure was observed in female GPER knockout mice only after 9 months of age (Martensson et al., 2009), which likely relates to the age-dependent decrease in endogenous estrogen and/or ERα and ERβ. Interestingly, in humans a mutant allele P16L which confers a single nucleotide polymorphism of GPER producing a hypofunctional phenotype is associated with significantly greater systolic blood pressure in female but not male carriers (Feldman, 2014; Feldman et al., 2014). G-1 reduces atherosclerotic lesion size in ovariectomized mice and lesion size is increased in the aortas of intact and ovariectomized female GPER knockout mice (Meyer et al., 2014). However, there is some evidence that GPER co-operates with ERα/ERβ in a larger signaling complex. Also, ERα and ERβ can exist as homodimers or heterodimers in the PM of endothelial cells (Levin, 2003). The importance of the constituents of the ER receptor complex and their role in the protective actions of estrogen will be discussed further throughout this review.

### 2.3 GPER1 in the heart and in cardiovascular disease

The effects of estrogen on cardiovascular physiology and CVD in general has been extensively reviewed in the last ten years (see for instance (Novella et al., 2019; Iorga et al., 2017; Murphy, 2011; Menazza and Murphy, 2016; Zimmerman et al., 2016; Groban et al., 2019)). However, due to the broad effects of ERs on a number of physiological and pathophysiological parameters which impinge on cardiovascular health, including glucose homeostasis, lipid homeostasis, vascular function, blood pressure and atherosclerosis, it is difficult to determine direct effects on the heart itself. Likewise, global knockout mice, particularly of GPER1, will affect all or some of these systems. Interestingly, some studies have shown effects of GPER1 activation on Ca^2+^ handling in cardiomyocytes, ie, inhibition of Ca^2+^ influx and decreased myofilament sensitivity to Ca^2+^ (Haas et al., 2009). Also, ERβ or GPER1 activation opens L- and R-type voltage gated Ca^2+^ channels in hypothalamic neurons (Sun et al., 2010; Micevych and Christensen, 2012; Mermelstein, 2009) and GPER1 activation leads to IP_3_ generation in breast tumour cells (Qian et al., 2016; Szatkowski et al., 2010), so although GPER effects may be Ca^2+^-dependent, the mechanism may be different in different cell types.

One way to study the effects of GPER1 on the myocardium directly is by using cardiomyocyte (CM) specific KO of GPER1 in mice. This leads to profound adverse cardiac remodeling and diastolic dysfunction in both male and female mice, with sex-based differences in gene expression profiles (Wang et al., 2017). Interestingly, GPER1 knockout affected left ventricular morphology and dimensions more in male mice. Analysis of DNA microarray data revealed pathway enrichment for genes involved in mitochondrial metabolism in females and inflammatory response genes in males. This suggests that the worse myocardial remodeling in males may be due to increased basal inflammation. Together with the protective effect of the GPER agonist G-1 against LV dysfunction and remodeling following ischemia-reperfusion, hypertension, high-salt diet or loss of estrogen (Deschamps and Murphy, 2009; Wang et al., 2012; Alencar et al., 2017; Bopassa et al., 2010; Filice et al., 2009; Jessup et al., 2010; Weil et al., 2010), these studies confirm the protective role of GPER1 in the heart. The mitochondrial gene expression alterations in female mice may relate to oxidative stress. A subsequent study in CM-specific GPER1 knockout female mice showed increased levels of oxidative stress and oxidant damage together with cardiac hypertrophy, fibrosis, increased left ventricular end diastolic pressure (LVEDP) and decreased fractional shortening. Interestingly, these changes were reversed by the mitochondrial-targeted antioxidant MitoQ (Wang et al., 2018).

Also, the role of GPER in immune cells is of particular interest and will impinge on CVD. GPER is expressed in many cells of the immune system, including lymphocytes, monocytes/macrophages, eosinophils and neutrophils (Notas et al., 2020). Estrogen inhibits monocyte/macrophage activation and therefore will affect sexual dimorphism in aspects of CVD such as atherogenesis. Whilst this has in part been attributed to ERα and ERβ, GPER1 mediates anti-inflammatory effects of estrogen in this cell type. E2 and G-1 decrease Toll-like receptor 4 (TLR4) expression in RAW 264.7 ​cells, primary mouse peritoneal macrophages, human monocytes and *in vitro* differentiated human macrophages, and decreases LPS induced expression of prostaglandin E_2_ (PGE_2_), IL-6 and TNFα (Rettew et al., 2010; Pelekanou et al., 2016). Interestingly, this appears to be due to a physical interaction with the ERα splice variant ERα36 and the p65 subunit of NFκB. Expression and colocalization of GPER1 and ERα36 was found in macrophages in atherosclerotic plaques in human coronary arteries from heart disease patients (Notas et al., 2020). GPER1 deficiency advances atherosclerosis progression in mice mainly by infiltrating immune cells in the vascular wall and mediated by inflammatory prostanoid production (Meyer et al., 2015). However, GPER1 activation may induce a pro-inflammatory response in neutrophils. G-1 increased the cytokines IL-1β, CXCL8 and increased cyclooxygenase-2 (COX-2) expression (Rodenas et al.G, 2017). Therefore, the effects of GPER1 activation may be cell-type or context specific amongst different immune cells. However, one caution is the possible off-target effects of G-1. Notwithstanding, taken together GPER1 appears to be protective in a vascular and a cardiac context.

## 3 Estrogen and the regulation of vascular tone

Cardiovascular disease in general is associated with the loss of estrogen and ERs with aging, especially post-menopause and therefore estrogen-dependent responses are considered to be a mechanism of protection against CVD and cancer. Given that estrogen receptor isoforms have differing binding affinities for E2 (Lin et al., 2013), and isoform distribution changes during and after menopause, this combined with decreased levels of circulating E2 may contribute to the relative loss of estrogen protective responses. This leads to hypertension, endothelial dysfunction and high circulating levels of cholesterol. To understand the causes of increased CVD prevalence in post-menopausal women, it is essential to comprehend the mechanism underlying E2 actions in pre-menopausal women (Kockx and Herman, 2000).

Vascular tone represents the level of blood vessel constriction in relation to its maximum diameter when dilated. Both the endothelium and the vascular smooth muscle in the vessel take part in maintaining the vascular tone. Furthermore, extrinsic and intrinsic factors also determine the extent to which a vessel relaxes/constricts. Extrinsic factors (such as circulating hormones; sympathetic nervous system) can alter the systemic (peripheral) resistance of the vasculature, thus modifying arterial pressure. Intrinsic factors (such as autocrine and paracrine mediators: CO, NO, histamine, neuropeptides, etc, and sheer stress via eNOS/NO) are primarily involved in maintaining/altering local blood flow within an organ. E2 is primarily an example of an extrinsic factor ie circulating gonadal E2 mainly responsible for its effects. However, autocrine regulation in hippocampal neurons by locally produced E2 (Prange-Kiel et al., 2003), the regulation of luteinizing hormone (LH) production in ovarian follicle granulosa cells by local estrogens (Kessel et al., 1985), and autocrine regulation of cell proliferation in estrogen-dependent breast cancer cells via ERα (Tan et al., 2009), are examples of intrinsic actions.

Furthermore, the conversion of androgens such as testosterone to estrogen by estrogen synthase (aromatase) is an important source of E2 in local tissues and is particularly important in males (see (Mendelsohn and Rosano, 2003) (Iorga et al., 2017)). Aromatase is found in vascular tissue, in both ECs and VSMCs and is widely distributed in extragonadal sites such as bone, brain, adipose tissue, including perivascular adipose tissue (PVAT) and blood vessels (Bulun et al., 2003; Harada, 1999). Aromatase is important in the cardiovascular system including coronary arteries (Diano et al., 1999). The use of aromatase inhibitors in women to treat breast cancer comes with significantly increased cardiovascular disease risk (Khosrow-Khavar et al., 2020). PVAT plays an important protective role in the maintenance of function of normal blood vessels, and its dysregulation in disease, such as in central obesity, it becomes proinflammatory and contributes to atherosclerosis (Brown et al., 2014; Qi et al., 2018). Local production of estrogen by PVAT plays a role in its homeostatic protective function (see for instance (Costa et al., 2018)). PVAT produces PVAT-derived relaxing factors (PVRFs), which activate VSMC ATP-dependent K^+^ channels (Lohn et al., 2002) and voltage dependent (Kv) K^+^ channels, such as KCNQ (Kv7) (Schleifenbaum et al., 2010). Because (PVAT-derived) E2 acts in a similar manner on VSMC relaxation and growth inhibition, it perhaps could be considered a PVRF (see for instance (Costa et al., 2018; Miao et al., 2011)).

### 3.1 Endothelial cells and Nitric Oxide (NO) production

NO is a molecule synthesized by the vascular endothelium from L-arginine via eNOS and is considered to be an intrinsic vasodilating factor. Vascular relaxation is only one of the multiple roles of NO and it is achieved through the activation of soluble guanylate cyclase (sGC), an enzyme that catalyzes the reaction which forms cyclic guanosine monophosphate (cGMP), which in turn activates protein kinase G (PKG). PKG can induce relaxation by triggering a decrease in intracellular Ca^2+^ by stimulating both the plasma membrane Ca^2+^ extrusion pump and the sarcoplasmic reticulum Ca^2+^ uptake pump (White et al., 1995) as well as via phosphorylation of myosin phosphatase subunit target 1 (MYPT1) which antagonizes the actions of Rho kinase and activation of myosin light chain phosphatase (MLCP) (Kaneko-Kawano et al., 2012) and phosphorylation of K^+^ channels which induce hyperpolarization (Zhou et al., 2010).

NO also inhibits VSMC proliferation and is well known for its antiplatelet, antithrombotic and anti-inflammatory actions. However, NO can also inhibit cytochrome C oxidase (CcOX), in complex IV of the mitochondrial electron transport chain, in competition with oxygen, and therefore cellular respiration. However, this is highly dependent on the respiratory state of the cell, the prevailing oxygen concentration and electron flux (reducing equivalents) (Palacios-Callender et al., 2007; Taylor and Moncada, 2010; Sarti et al., 2012). For instance, under hypoxic conditions, with an increasing proportion of cytochrome C oxidase (CcOX) in the reduced state. This is an adaptive response, and thus under low O_2_ concentration conditions the resulting increased bioavailability of NO locally can activate soluble guanylate cyclase (sGC) resulting in vasodilatation and therefore the local supply of O_2_ (Palacios-Callender et al., 2007). Under certain conditions (ie hypoxia, or at least when NO concentration exceeds O_2_ concentration and reduction of CcOX occurs) this could (at least in theory) lead to peroxynitrite (ONOO^−^) radical formation (a strong oxidant species formed from the reaction of NO and superoxide-O_2_^-^) due to increased O_2_^−^ generation. This is due to the leak of electrons from complexes I and III when O_2_ is not completely reduced to H_2_O (Taylor and Moncada, 2010). It has been proposed that this may be a mechanism associated with vascular aging and disease (Wink and Mitchell, 1998). However, this idea remains controversial, because other oxidoreductase enzyme systems in ECs and VSMCs may, in fact, contribute more to O_2_^−^ generation, such as PM NADPH oxidases (NOXs) and uncoupled eNOS, particularly under normoxic conditions and in response to GPCR agonists and stretch. Notwithstanding the source of vascular O_2_^−^ and ONOO^−^ formation, there is evidence that E2 reduces the formation of these by inducing the transcription of superoxide dismutases SOD1 and SOD2 (a genomic effect) (Strehlow et al., 2003), and/or has direct radical scavenging properties (Prokai and Simpkins, 2007) as well as reducing production of the pro-inflammatory cytokines TNFα and IL-1β (Straub, 2007; Novella et al., 2012). Therefore, E2 plays a major role preventing vascular aging and disease. This may underlie not only the cardioprotective effects of GPER activation against I/R injury (ie by G-1) (Deschamps and Murphy, 2009), but also the inhibitory effects of ERα and GPER activation on VSMC proliferation post-injury (Pare et al., 2002; Li et al., 2013). However, both pro- and anti-inflammatory effects of E2 have been reported (see for instance (Novella et al., 2012)). Interestingly, this may be dependent on relative changes in the levels of ERα versus ERβ, since an age-related increase in ERβ is associated with a pro-inflammatory profile of E2 (Novella et al., 2012).

E2 initiates the production of NO by activating eNOS, both in a Ca^2+^ independent and a Ca^2+^-dependent manner via ERα, ER46 or ERβ, depending on the vascular bed. E2 can promote NO production in two ways: via Mitogen Activated Protein Kinase (MAPK-ERK1/2) and via PI3K/Akt, but how these mechanisms occur independently of Ca^2+^ increase, is still not completely understood. Russell et al. (2000) demonstrated that E2 facilitates Hsp90 association with eNOS (an important association because Hsp90 acts as a scaffold and aids the phosphorylation of eNOS by Akt), thus reducing Ca^2+^ requirements of eNOS. By using geldanamycin, a drug that binds the ATP binding region of Hsp90 and inhibits its action, NO could not be released under the stimulation of E2, showing the importance of Hsp90 in the E2 action on eNOS.

### 3.2 The phosphoinositol-3-kinase (PI3K)-Akt-eNOS pathway

**Fig. 3:**
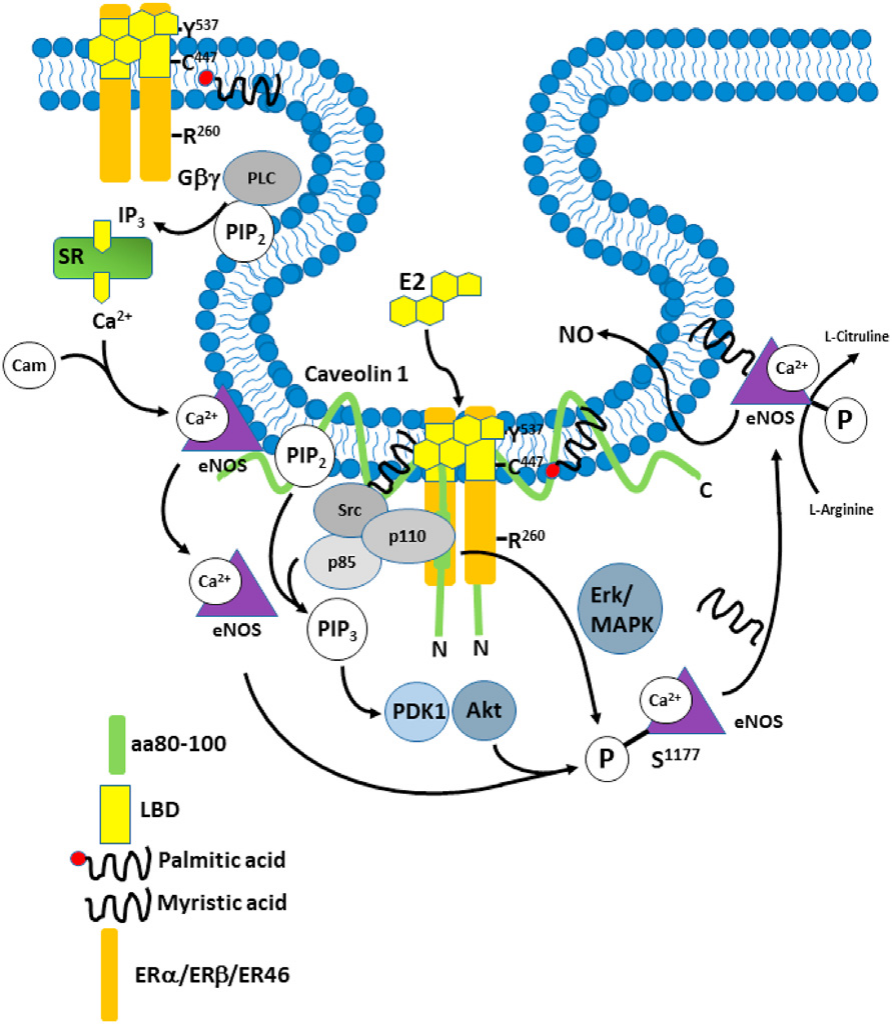
Production of NO after E2 binding. A functional signaling complex containing c-Src, PI3K and ER46 co-localized with Caveolin-1 in ECs. Membrane localization requires palmitoylation of ER46 at Cys^447^ and also lipid modification of c-Src. ERα membrane localization also requires palmitoylation at Cys^415^. Gβγ activation of phospholipase C generates IP_3_ and diacylglycerol from membrane PIP2. IP_3_ causes Ca^2+^ release from the endoplasmic reticulum which binds to calmodulin (Cam) which then binds to eNOS and causes eNOS to dissociate from caveolin 1 and come off the membrane. Activation of PI3K leads to the conversion of PIP2 to PIP3 which activates PDK1 and Akt. Along with MAPK (Erk1/2) activation this phosphorylates eNOS on Ser^1177^ and translocates it back to the plasma membrane following myristylation, where it catalyzes the formation of NO and L-citrulline from L-arginine. NO release induces VSMC relaxation. Adapted from (Orshal and Khalil, 2004).

After it was demonstrated that Akt, the downstream target of PI3K, phosphorylates eNOS and potentiates Ca^2+^ and calmodulin association (Fulton et al., 1999), Haynes et al. (2000) used human umbilical vein endothelial cells (HUVECs) and EA hy926 (human endothelial-like) cells to evaluate E2’s ability to activate Akt by binding cellular membrane receptors. The cells were incubated with either E2 alone, LY294002-an inhibitor of PI3K, ICI182,780-a non-specific ER antagonist or ionomycin-an ionophore used to raise intracellular levels of Ca^2+^. When E2 was administered there was a 4-fold increase in NO. Neither LY294002 nor ICI182,780 affected the basal levels of NO (tonic levels of NO produced by the endothelium) but abrogated the E2 induced NO increase. This shows that E2 can significantly increase NO release in ECs via an ER-mediated pathway in a PI3-kinase-dependent manner. By using a non-selective ER antagonist, activation of all estrogen receptors was inhibited. Therefore, this study cannot indicate the specific receptor that produced the vasodilatory actions, but we can presume that it is either ERα or ERβ (as depicted in Fig. 3) because of the Akt-PI3K association with ER46 and due to the ability of ERβ to also trigger the activation of this pathway. A functional signaling complex containing c-Src, PI3K and ER46 co-localized with Caveolin-1 in ECs has been demonstrated (Li et al., 2003, 2007; Haynes et al., 2003; Kim and Bender, 2005). Membrane localization requires palmitoylation of ER46 at Cys^447^ and also lipid modification of c-Src. ERα membrane localization also requires palmitoylation at Cys^415^ (Adlanmerini et al., 2014). E2 was reported to stimulate proliferation via activation of PI3K via ERα but not ERβ in breast cancer cells (Lee et al., 2005), although this was secondary to increased PI3K p85 subunit expression. Nevertheless, rapid activation of eNOS in EA Hy926 ​cells was PI3K- and ER-dependent (Hohmann et al., 2016). Activation was blocked by ICI 182,780 which would appear to implicate ERα coupling to PI3K. However, as mentioned above, ICI 182,780 also activates GPER.

Nevertheless, a direct association between ERα rapid, membrane delimited signaling and PI3K has been demonstrated in ECs (Simoncini et al., 2000, 2003) and breast cancer cells (Castoria et al., 2001) and ERα binds directly to p85 (Simoncini et al., 2000) (see also (Menazza and Murphy, 2016)). A more definitive association was confirmed from individual knockout studies. It was shown that ERα mediates endothelial NO production and anti-atherosclerotic effects (Chambliss et al., 2000; Meyer et al., 2014; Kim et al., 2014). Likewise, ERα rapid signaling is required for E2 induced proliferation and migration of ECs (Lu et al., 2016), as shown using a triple (KRR) mutant of ERα which is specifically defective in rapid signaling but leaves the ERα genomic signaling function intact. PI3K-dependent Akt phosphorylation was also defective in the KRR mutant ECs. Reduced PI3K activation has also been observed in ERβ knockout hearts (Wang et al., 2009) and hypothalamic neurons (Gingerich and Krukoff, 2008). It therefore seems reasonable to assume that both ERα and ERβ can activate PI3K in a similar, direct, membrane delimited manner. In contrast, GPER appears to activate PI3K indirectly, via transactivation of the EGFR (see for instance (Prossnitz and Barton, 2014)).

eNOS is largely regulated by cytosolic Ca^2+^ and calmodulin. Estrogen binding to membrane ER in various cell types induces Ca^2+^ mobilization with rapid kinetics. Particularly in neuronal cells this can be mediated by several types of PM voltage operated or store operated Ca^2+^ channels (see (Rybalchenko et al., 2009)). Indeed, activation of PM receptors by E2 results in the formation of IP_3_ and cAMP and therefore stimulates Ca^2+^ release from the endoplasmic reticulum and also E2 induces Ca^2+^ transients in HUVECs (Caulin-Glaser et al., 1997), although in ECs this is probably via store operated channels, as they don’t have voltage operated channels. Interestingly, Rybalchenko et al. (2009) showed that ERβ can induce RyR2-dependent Ca^2+^ release from the endoplasmic reticulum by direct interaction in the absence of ligand (E2) binding in HT-22 neuronal cells. However, the situation in vascular cells is less clear, but RyR2/3 in ECs could possibly amplify IP_3_-induced release via CICR. The CAM complex is formed by Ca^2+^ binding to calmodulin and aids the activation of eNOS by dissociating it from the caveolin 1 complex (Goetz et al., 1999), as depicted in Fig. 3. However, in another study, cytosolic levels of Ca^2+^ were measured by fluorometric assay in response to E2 and histamine administration. The experiment demonstrated the expected intracellular Ca^2+^ increase but estradiol alone did not promote HUVEC Ca^2+^ fluxes at any concentration, although it still rapidly (10 ​min) induced an increase in NO release. Thus, in E2 (alone) mediated NO release, a rise in intracellular Ca^2+^ is not required even though E2 can elicit a Ca^2+^ transient (Caulin-Glaser et al., 1997). Despite this, there is some evidence that by phosphorylating eNOS, Akt can activate the enzyme at much lower Ca^2+^ and calmodulin concentrations. This is thought to be caused by a faster electron flux that passes across the eNOS reductase domain and due to a lower rate of calmodulin dissociation from eNOS at low Ca^2+^ levels (McCabe et al., 2000), similar to the effect of Hsp90.

The extent to which ER and GPER signaling is IP_3_/Ca^2+^-dependent may depend on the relative distribution of the receptors in ECs and VSMCs and also differences between vascular beds. For instance, vasodilation induced by selective GPER agonists (ie G-1) in different vascular beds, ie, female mesenteric resistance arteries, requires endothelial NO and smooth muscle cAMP (Lindsey et al., 2014). In rat carotid, GPER induced vasodilation is endothelium-dependent, rather than endothelium independent and VSMC-driven (Broughton et al., 2010). In coronary arteries the data is conflicting, because G-1-dependent relaxation has been shown to be endothelial NO-dependent in porcine coronary arteries because it was abolished by L-NAME or endothelial denudation (Meyer et al., 2010). However, in other studies relaxation to G-1 still occurred in endothelium denuded aorta or coronary arteries (Lindsey et al., 2009; Yu et al., 2011). GPER activation does increase intracellular Ca^2+^ mobilization, but in some cells this may be due to EGFR transactivation rather than a PLCβ-IP_3_ -mediated mechanism (Revankar et al., 2005), because EGFR can activate PLCγ-IP_3_. However, this was in fibroblasts where GPER is localized to endoplasmic reticulum and Golgi rather than the PM. In vascular cells, GPER activation can lead to endothelium-dependent and endothelium independent mechanisms, as mentioned above. Therefore again, responses may differ between different vessels and different vascular beds. As mentioned previously, the VSMC-relaxing effect of GPER activation may be mediated via a Ca^2+^-activated large conductance K^+^ channel (BKca) (Yu et al., 2011), therefore presumably involves Ca^2+^ mobilization or changes in BKca Ca^2+^ sensitivity, for instance via PKG-dependent phosphorylation. Given that increased intracellular Ca^2+^ induces VSMC contraction, this perhaps suggests a localized Ca^2+^ pool at the PM which regulates BKca. Indeed, others suggest that E2 induces VSMC relaxation by interfering with Ca^2+^ mobilization and entry (Crews and Khalil, 1999a, 1999b; Mazzuca et al., 2015). Furthermore, activation of K^+^ channels would lead to membrane hyperpolarization and therefore relaxation via feedback inhibition of VOCC (see (Novella et al., 2019; White, 2002)). In female rat mesenteric micro-vessels, ER (mainly ERα) directly mediates decreased VSMC Ca^2+^ entry via endothelium- and K^+^ channel-independent mechanisms (Mazzuca et al., 2015).

Rapid signaling by ERα and ERβ mainly involves kinase activation at the membrane, via PI3K, ERK (MAPK) and eNOS activation in ECs and impinges on proliferation and migration, as well as vasodilation (Guo et al., 2005). ERα also activates Gαi and Gβγ (Kumar et al., 2007), which is also involved in eNOS and ERK activation (Guo et al., 2005), seemingly via Gαi induced activation of c-Src (Li et al., 2007). This pathway contributes to the stimulatory effect of ERα activation on EC proliferation and migration (Chambliss et al., 2010; Ueda et al., 2013). However, ERα activation in human and rat ECs modulates intracellular Ca^2+^ and causes a rapid increase in intracellular Ca^2+^ which is blocked by the ERα antagonist ICI 182,780 (Stefano et al., 2000; Rubio-Gayosso et al., 2000). This effect is presumably mediated via Gβγ-dependent IP_3_ -mediated Ca^2+^ release.

ERα and ERβ signaling is similar in VSMCs where activation rapidly inhibits proliferation. However, in the case of VSMCs the balance appears to be in favour of phosphatase rather than kinase activation, ie, ERα activation stimulates increased activation as well as expression (ie a genomic effect) of the phosphatases MKP-1, PTEN, PP2A and SHP-1, which inhibits PI3K and ERK activation and reduces proliferation and migration (Menazza and Murphy, 2016; Li et al., 2011; Lu et al., 2003; Yang et al., 2012). ERα membrane recruitment and activation is dependent on an interaction with the scaffold protein striatin. In transgenic mice where this interaction is inhibited (Zheng et al., 2018), or overexpressing an ERα trafficking inhibitory peptide (Ueda et al., 2013) the E2-depedent inhibition of VSMC proliferation and migration is lost.

### 3.3 The mitogen-activated protein kinase (MAPK)-eNOS pathway

The MAPKs are serine threonine kinases and one of their subcategories is represented by the extracellular-signal regulated kinases (ERKs). ERKs can be activated by the Ras-Raf-MEK cascade. E2 binding to ERα or ERβ leads to GTP loading of Ras and activation. Ras then recruits Raf kinase which activates MEK1 (MAPK/ERK kinase or mitogen-activated protein kinase kinase (MAPKK)). In turn, MEK1 will phosphorylate ERK 1/2. To investigate whether E2 activates this pathway, Chen et al. (2004) analyzed how 17βestradiol affects the phosphorylation of ERK1/2 in steroid-starved UAECs. Treatment for 10 ​min with physiological concentrations of estradiol (10 nM-1 μM) caused the rapid phosphorylation of ERK1/2 up to 6-fold the basal level after 5 ​min and the maximal response was then maintained for up to 60 ​min. Furthermore, to show that the ERK1/2 upstream activator Raf-1 is also activated by E2 in ECs, an immunocomplex kinase assay was carried out. The assay revealed an ordered activation of Raf-1, MEK1, and ERK2, with a 30% (above control) increase in Raf-1 activity after administration of 10 ​nM E2 (10 ​min). When pretreated with a MEK1 and MEK2 inhibitor (PD98059), E2 treated UAECs showed reduced eNOS activity, demonstrating that the MAPK pathway has a high importance in mediating E2 induced eNOS activity. Results using E2-BSA, a membrane impermeable estradiol conjugate, to test whether the ER involved in eNOS activation is a membrane receptor (i.e. non-genomic rapid signaling), were essentially the same. However, a possible caveat of using 17β-estradiol-BSA is that it was reported to be susceptible to contamination with 17β-estradiol and as a consequence it could produce inaccurate results (Taguchi et al., 2004).

The upstream activation of Ras by E2 binding is less well documented, but it is thought that activation of c-Src (Fig. 2) is one of the initiating steps of this signaling cascade. E2-induced MAPK activation was abrogated by treatment of HUVECs with a c-Src selective inhibitor (Klinge et al., 2005). GTP bound Ras can also bind and activate PI3K, thus further potentiating the action of E2 (Haynes et al., 2003). However, these 2 studies did not investigate the interaction between ERα and Gαi, a mechanism that is fundamental for both c-Src and ERK activation. Also the Gβγ subunit facilitates ERα interaction with Gαi. This mechanism is independent of GPCR activation (Kumar et al., 2007) (and see (Menazza and Murphy, 2016)). Gαi and Gβγ directly interact with ERα via two regions-aa251-260 and aa271-595 respectively, which leads to eNOS activation. Furthermore, E2 induces the release of Gαi and Gβγ without GTP binding to Gαi. Disruption of the Gαi interaction with ER by mutation of these regions or using blocking peptides, or of Gβγ to the β-adrenergic receptor kinase (βARK) blocked the non-genomic response to E2 and also downstream c-Src and ERK activation in Cos7 cells (another fibroblast cell line). In ECs, disruption of ERα and Gαi interaction blocked E2-induced eNOS activation and also attenuated monocyte adhesion (Kumar et al., 2007). Other studies in Cos-7 ​cells expressing ERs have shown that overexpression of ERβ in the membrane caveolae also induces rapid eNOS activation, independently of ERα, which suggests that ERβ can also mediate the rapid effects of E2 on eNOS (Muka et al., 2016). The ERβ signaling complex is similar ie involving Gαi, Gβγ, c-Src and PI3K.

### 3.4 Involvement of GPER1 in endothelium-dependent and -independent vasodilation

The constriction and relaxation of smooth muscle cells is mainly managed by paracrine or autocrine factors, by hormones and by stretch (myogenic response). VSMCs can also respond to changes in load by tonic and phasic contractions. To initiate contraction, VSMCs require myosin and actin cross-bridge formation and an increase in cytosolic Ca^2+^. Ca^2+^ increases in response to stimuli such as endothelin-1 and angiotensin II and forms a complex with Calmodulin (CaM). This complex can activate Myosin Light Chain Kinase (MLCK), which will phosphorylate the myosin light chain. The increase in cytosolic Ca^2+^ is due to both its release from the sarcoplasmic reticulum and because of it entering the cell from the extracellular space via Ca^2+^ channels. Voltage gated (L-type) Ca^2+^ channels open in response to agonists or stretch which cause depolarization of the membrane. Furthermore, they can also open when they are phosphorylated by protein kinase C (PKC). PKC activation occurs as a result of agonist binding to GPCRs that are coupled to a heterotrimeric G protein. The Gβγ subunit stimulates phospholipase C activity which catalyzes the formation of IP_3_ and diacylglycerol (DAG) from the membrane phospholipid PIP_2_. By binding sarcoplasmic reticulum IP_3_ receptors, IP_3_ triggers Ca^2+^ release into cytosol. The existing Ca^2+^ and DAG then activate Ca^2+^-dependent PKC isotypes (ie, PKCα) which further aids the increase of intracellular Ca^2+^ by phosphorylation of the L-type Ca^2+^ channel (Kim et al., 2008).

GPER has been shown to trigger the activation of rapid signaling pathways when bound by E2. Confocal microscopy studies localized GPER primarily to the endoplasmic reticulum, although it was presumed to be localized at the plasma membrane. One explanation for this apparent discrepancy might be that after agonist stimulation, or during receptor biogenesis, GPCRs traffic between the endoplasmic reticulum and the plasma membrane (Revankar et al., 2007; Kleuser et al., 2008) (and see section 2.2 above). Also, possible, although not substantiated, is that GPCRs can traffic between the PM and endosomes and receptors can signal from endosomes in complexes similar to caveolae. GPER activation separately leads to both increases in cAMP and also triggers c-Src with opposing effects on relaxation or contraction, ie, c-Src is involved in EGFR transactivation via the activation of matrix metalloproteinases (MMPs), which will in turn lead to the MAPK and PI3K cascade activation and vasoconstriction in VSMCs. However, GPER activation in ECs leads to E2-induced NO release and relaxation. Also, GPER-dependent cAMP activation in VSMCs leads to vasorelaxation in coronary arteries. How do these conflicting pathways co-exist ?

GPER activation can lead to both relaxation or contraction of coronary arteries depending on the specific conditions. This apparent contradiction may be due in part to direct GPER-dependent relaxation in ET-1 pre-constricted porcine coronary arteries (ie EC-dependent via eNOS?), compared to GPER-dependent EGFR transactivation in VSMCs causing constriction (Yu et al., 2018). In this scenario the GPER agonist G-1 caused relaxation of ET-1 pre-constricted arteries which was blocked by the GPER antagonist G36. However, G-1 pre-treatment enhanced the ET-1 dependent constriction (ie if given before ET-1). This was blocked by the EGFR antagonist AG4178 or inhibition of c-Src. Also, this enhanced relaxation, suggesting that these two mechanisms are in opposition in PCASMCs. Furthermore, the G-1 enhanced ET-1-induced constriction was blocked by the ERK inhibitor PD98059, which also enhanced relaxation. However, since ERK is also involved in eNOS activation, the net effect of ERK inhibition on vascular tone will depend on the experimental conditions, such as viability of the ECs *in vitro*, the choice of pre-constrictor agent and perhaps more importantly the dose of pre-constrictor used. Also, intriguingly inhibition of Gβγ with gallein inhibited G-1 enhanced constriction and potentiated relaxation (Yu et al., 2018). However, although this study didn’t directly test whether the G-1-induced relaxation was Gα or cAMP-dependent, previous work from the same group reported that GPER-dependent coronary artery relaxation was due to cAMP/PKA-dependent phosphorylation and activation of myosin light chain phosphatase (MLCP) in VSMCs, which was in itself Gαs-dependent rather than Gαi (Yu et al., 2014).

These apparently opposing responses are likely dependent on the relative distribution of GPER between ECs and VSMCs. For instance, if EGFR activation of c-Src and ERK/MAPK causes constriction in VSMCs, how does this then relate to eNOS-dependent NO production in ECs ? The rapid effects of E2 on VSMCs which induce vasodilation also occurs in endothelium-denuded vessels of rabbit and human coronary arteries that were treated with ET-1. This implies that the relaxation caused by E2 in VSMCs is independent of E2’s actions on the endothelium (Jiang et al., 1992).

A surprising finding with possible clinical significance was that ICI182,780, an antagonist of ER, (and other antagonists such as tamoxifen, raloxifene and phytoestrogens) are agonists for GPER (Thomas et al., 2005). This finding highlights the importance of taking into consideration all 3 types of receptors in experiments studying both ECs and VSMC, something that was not done in the experiments above. For example, by administering ICI182,780, GPER stimulated pathways would be activated and classical ERs pathways inhibited.

### 3.5 Contradictory effects of GPER1 activation

Before the discovery of GPER1, it was thought that only ERα and ERβ are involved in VSMC relaxation. In contrast, knockout mice with no classical ERs, still had vasodilatory responses to E2 administration. GPER1 was found to be expressed at high levels in arterial VSMCs, the arteries of humans with coronary atherosclerosis (Haas et al., 2007) and in the arteries of hypertensive mRen2 Lewis rats (Lindsey et al., 2009). Furthermore, by injecting G-1, a GPER1 agonist, in rats with normal blood pressure, a marked reduction in MABP was noticed within 2 ​min after infusion. By measuring changes in the lumen diameter of pre-constricted rat mesenteric arteries over time, after the administration of the same agonist, acute dilation was recorded. Interestingly, the effect of G-1 in human internal mammary arteries were greater than that of E2, with G-1 exerting a stronger relaxant response. In contrast to G-1, 17β-estradiol only dilated murine aortas, but not murine carotid arteries (Haas et al., 2009), which suggests how different and varied the distribution of estrogen receptors is throughout the vasculature. Additionally, GPER1 knockout mice, had increased blood pressure due to increased peripheral vascular resistance as manifested by increased media to lumen ration in resistance arteries as well as hyperglycaemia, reduced glucose tolerance and skeletal/growth defects in female mice, emphasising the important metabolic roles of GPER1 (Martensson et al., 2009).

In contrast to expectations, Kurt and Buyukafsar (2013) found that when isolated perfused rat kidney arteries were treated with G-1, in the absence of vasoconstrictors, they experienced a substantial vasoconstriction. E2 treatment had similar but weaker effects, while PPT, the ERα agonist had no effects over the perfusion pressure and neither did DPN, the ERβ agonist, showing that the mechanism is or can be independent of the classic estrogen receptors. Furthermore, G-1 vasoconstriction was not modified by endothelium denudation, showing that none of the endothelial factors are able to compensate for the vasoconstriction caused by GPER1 activation. To clarify that GPER1 was responsible for the actions, G15, its antagonist was used in the same preparation after G-1 treatment and it significantly abrogated the vasoconstrictor response. ERK1/2 inhibitors also blocked E2 and G-1 effects, suggesting the involvement of this pathway in VSMCs constriction. Interestingly, in the same arteries that were pre-constricted using different factors, G-1 acted as a vasodilator. This shows that the effects may be different in vessels isolated from different vascular beds and that the results depend on experimental set-up (ie whether they possess pre-existing tone or have been pre-constricted with agonists). The mechanism proposed by Yu et al. (2018), could explain this contradiction, and some of the conflicting results obtained from HRT studies and the question that arises from the study by Kurt and Buyukafsar (2013) as to why endothelium denuded renal arteries that were not treated with ET-1, but only with G-1, still had vasoconstricting effects. Yu et al. showed that G-1 mediated relaxation in PGF2α pre-constricted porcine coronary arteries (PCAs) and porcine coronary artery smooth muscle cells (PCASMCs), is cAMP and PKA-dependent via decreased phosphorylation of the MLCP regulatory subunit MYPT1 (Yu et al., 2017). This occurred in both aortic rings and PCASMCs via downregulation of RhoA/Rho kinase activity and thus phosphorylation of MLC. Furthermore, the resulting PKA activation was A-kinase anchoring protein (AKAP)-dependent (Yu et al., 2014). They further showed that this occurs via Epac/Rap1-mediated inhibition of RhoA/Rho kinase in *parallel* with PKA-dependent phosphorylation of vasodilator stimulated phosphoprotein (VASP), which also inhibits Rho kinase. In a further investigation of the upstream signaling the same group showed that GPER activation by G-1 in PCAs and PCASMCs occurs via Gαs activation by forming a plasma membrane complex with AKAP and the membrane-associated guanylate kinase (MAGUK) SAP97 (Gonzalez de Valdivia et al., 2017). Interestingly, these relaxant effects of GPER activation also occurred in endothelium denuded coronary arteries, so were direct effects on CASMCs and endothelium-independent. Perhaps, the final piece in the jigsaw is the observation that whereas G-1 caused dose-dependent relaxation in ET-1 pre-constricted arteries, it enhanced ET-1 contraction when given before ET-1. Inhibition of Gβγ with gallein, EGFR with AG1478 or Src with PP2 blocked the constrictor effects of G-1 and enhanced the relaxant effects. Also, inhibition of ERK1/2 with PD98059 did the same. So GPER causes EGFR transactivation which causes ERK-dependent constriction via Gβγ -dependent Src activation (Yu et al., 2018). It could also be that there are other mechanisms that remain to be discovered or GPER plays different roles in different vascular tissues or GPER distribution between ECs and VSMCs has different effects, but these studies go a long way to explaining the paradoxical effects of GPER activation.

**Fig. 4:**
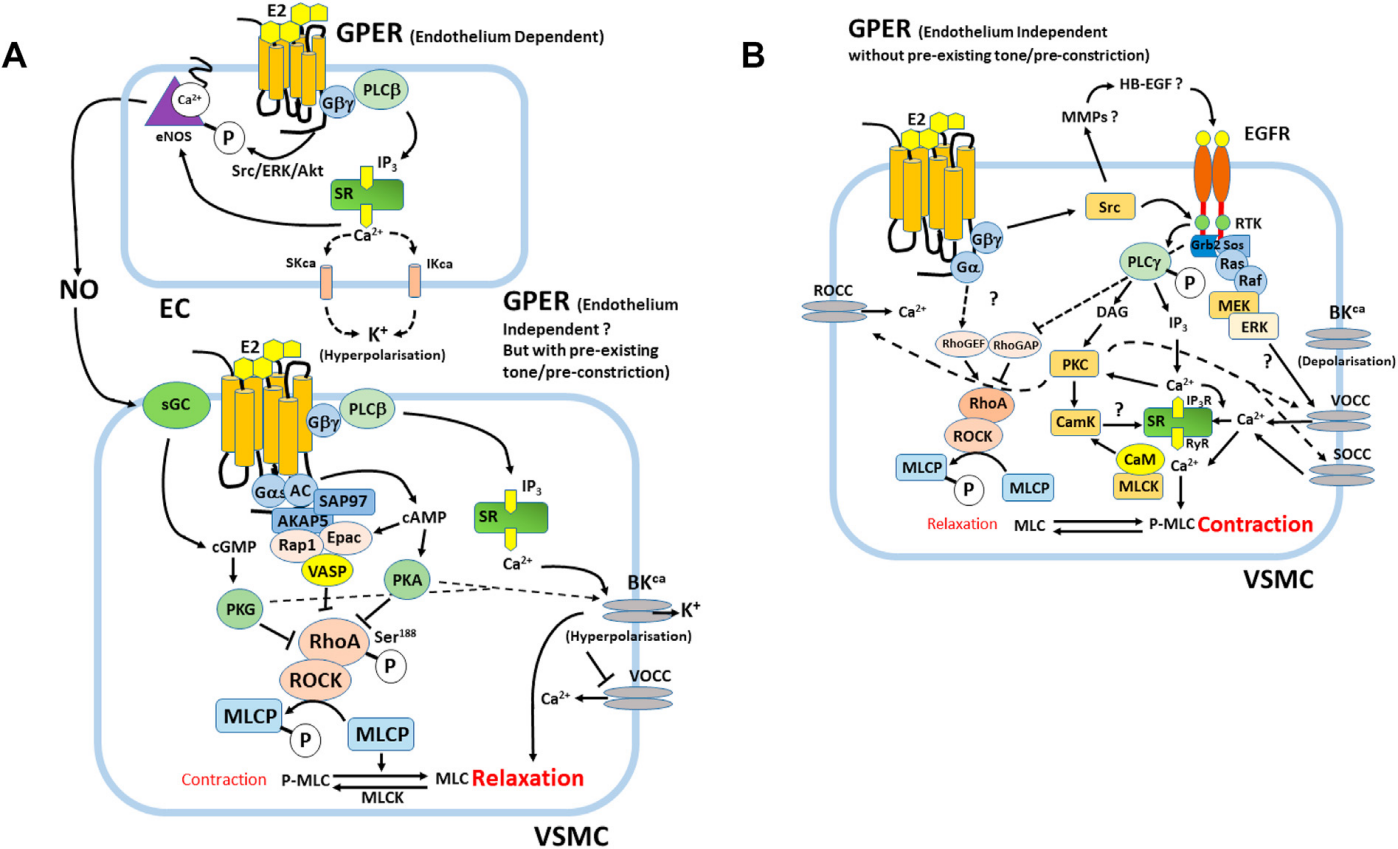
GPER-dependent signaling events in vascular endothelial and smooth muscle cells (VSMCs). **Panel A:** Endothelium-dependent (NO-dependent) and -independent modes of GPER-dependent relaxation in the presence of pre-existing vascular tone or pre-treatment with GPCR vasoconstrictors (Ang II or ET-1) or PGF2α. GPER agonists such as G-1 or E2 cause vasorelaxation when administered *after* pre-constriction. Note in this diagram, for simplicity, GPER is depicted as being in the PM, but in fact may signal intracellularly, ie from the endosomal membrane. **Panel B:** Endothelium-independent GPER-dependent vasoconstriction occurs in the absence of pre-existing vascular tone or when GPER agonists (G-1 or E2) are administered *before* vasoconstrictor agonists and probably involves Epidermal Growth Factor Receptor (EGFR) transactivation.

Despite the finding that GPER can produce vasoconstriction, studies on rat aorta, human mammary arteries and rat and porcine coronary arteries (lacking or having intact endothelium) concluded that its actions are predominantly vasodilating (Haas et al., 2009; Lindsey et al., 2009; Broughton et al., 2010; Yu et al., 2014), ie, if arteries have pre-existing vascular tone as they are likely to have *in vivo*. As mentioned above, one of the main ways to produce vascular contractions is via phosphorylation of myosin light chain (MLC) by MLCK (Kim et al., 2008). The finding that cAMP decreases this phosphorylation (Aslam et al., 2010) opened new horizons for investigating whether there could be a link between E2 and cAMP actions. As mentioned above, studies on porcine endothelium-denuded arteries, pre-constricted with PGF2α and then G-1 treated, showed a significant concentration dependent increase in cAMP production compared to the control group, and tension studies also confirmed G-1-dependent relaxation. PKA is a well-known target of cAMP and a very small concentration of G-1 raised PKA activity two-fold compared with the control, whilst G36 (GPER antagonist) completely blocked this effect. One of PKA’s targets is RhoA, which is inhibited by PKA phosphorylation. The Rho family of small GTPases are involved in multiple cellular mechanisms, one being switching off MLCP to allow for smooth muscle contraction. Inhibiting G-1 action using a PKI, decreases the phosphorylation of RhoA, whilst a PKA agonist in absence of G-1, produces similar effects to those of G-1 alone. This demonstrates that RhoA inactivation can be induced by G-1. In support of this general scheme, it was reported that cAMP/PKA also mediates relaxation in adult New Zealand white rabbit femoral arteries downstream of activation of GPER with G-1 (Porter et al., 2006). The probable mechanisms of GPER mediated vasodilation and constriction are outlined schematically in Fig. 4 panels A and B (and see (Yu et al., 2014)).

### 3.6 Ca^2+^- and voltage-dependent large conductance potassium channel (BKca) activation and VSMCs relaxation

BKca channels (large conductance calcium- and voltage-dependent K^+^ channels) are one of the main K^+^ channels present in VSMCs of human coronary and other arteries and have an important role in regulating membrane potential, in response to depolarization by vasoconstrictor molecules or stretch by causing an opposing hyperpolarization (see (Yang et al., 2013) and refs). Patch-clamped cultured human coronary artery smooth muscle cells, were treated with 17β-estradiol to study whether estrogen could activate these channels and therefore induce relaxation by increasing potassium efflux and closing VDCCs. The results indicated a concentration and time dependent stimulation of these channels, with maximal effects noticed 30 ​min after E2 administration, that persisted for 100 ​min on average (White et al., 1995). Although in this study these were supraphysiological concentrations of E2 (5 ​μM), physiological concentrations of E2 (100 pM-1 ​nM) also activate BKca (Nishimura et al., 2008).

Yu et al. (2011), showed increased channel opening by E2 treatment. Furthermore, in the same cells (E2 treated) this activation was blocked by the highly selective BKca channel blocker, iberiotoxin, supporting the conclusion that E2 opens BKca channels. It is well accepted that PKG depends on cGMP to activate myosin light-chain phosphatase (MLCP) which then leads to smooth muscle relaxation. Usually cGMP increases in response to sGC activation by endothelial NO. However, E2 stimulated VSMCs showed increased levels of cGMP and increased PKG activity, which meant that there is an association between cGMP, PKG and BKca channels. This was independent of NO since there were no endothelial cells present. Additionally, inhibition of PKG reversed the effect of E2 on BKca activity which suggests that E2 activation of BKca is PKG-dependent (White, 2002). Despite this finding, the exact mechanism through which E2 activates cGMP and PKG in the absence of NO is unclear. However, it is also likely that increased NO production in ECs in response to E2 also contributes to the cGMP-mediated BKca activation. Also, differences between different arterial beds in BKca β-subunit expression regulates BKca Ca^2+^ sensitivity and therefore the magnitude of the response to changes in intracellular Ca^2+^ (Yang et al., 2013). Also, E2 affects β-subunit expression in arterial smooth muscle (Nagar et al., 2005) and mouse uterus (Benkusky et al., 2002). Furthermore, induction of BKca subunit expression was ERβ-dependent in neuronal cells (Li et al., 2005) and possibly myometrium (see also (Lorca et al., 2014)). Although these are genomic effects, since BKca subunit expression declines with age, it is relevant for HRT studies.

More recent findings showed cAMP-dependent cross-crosstalk with PKG activation in VSMCs, which was GPER1-dependent. This is partly due to endothelial NO-dependent activation of sGC in VSMCs downstream of GPER and crosstalk with GPER-dependent AC activation in VSMCs themselves, which then potentiates PKG (Lindsey et al., 2014). Similarly, Keung et al. (2005) showed that administration of cAMP and cGMP antagonists to porcine coronary arteries pre-treated with 8-Br-cGMP (an activator of PKG) or estradiol, inhibited relaxation of the vessels. In this study PKA inhibition had no effect on estradiol treated arteries. However, other studies suggest that G-1 induced coronary artery vasodilation is due to Epac/Rap1 and PKA-dependent inhibition of RhoA/Rho kinase and activation of MLCP (Yu et al., 2017) (ie, see Fig. 4, panel A). Administration of both AC and GC inhibitors also abolished vasorelaxation in mesenteric arteries but only partially reduced vasodilation individually (Lindsey et al., 2014). Also, cAMP is a partial agonist for PKG (VanSchouwen et al., 2015). So, the contribution of GPER to artery relaxation is complex due to the expression of GPER on both endothelial cells and VSMCs which are differently coupled to downstream signaling and may therefore produce direct or indirect effects on VSMCs. Furthermore, and as mentioned above, Yu et al. showed that GPER induced relaxation involves recruitment and activation of Gαs and the guanylate kinase SAP97 which could also link cAMP and cGMP-dependent signaling (Gonzalez de Valdivia et al., 2017).

## 4 Other E2 actions important in maintaining vascular health

### 4.1 ERs in VSMC proliferation

Although membrane ERα and ERβ do not mediate the relaxing effects of E2 upon VSMCs, they are required for rapid estrogen-induced inhibition of VSMC proliferation, a mechanism that is thought to stop the progression of atherosclerosis and restenosis. The mechanism through which E2 inhibits VSMC proliferation is not known in detail. However, protein phosphatase 2 ​A (PP2A) activation is thought to play a role, due to its importance in regulation of cell metabolism and cell cycle (Ueda et al., 2013).

The importance of these 2 receptors in the protection from vascular injury was also studied in knockout mice with either no ERα or ERβ. Mice deficient in ERβ were shown to still retain vascular protective mechanisms to an extent, when E2 was administered (Karas et al., 1999). On the other hand, in ERα knockout mice, no inhibition of VSMCs proliferation was observed in response to vascular injury, showing the importance of ERα in this mechanism of action (Pare et al., 2002). However, these studies did not investigate whether the rapid non-genomic mechanism is sufficient for the inhibitory effects on VSMC proliferation. It might be that both nuclear and non-genomic actions are required and that there is a link between the two that remains to be investigated. Discovering the pathway would open new perspectives in targeting specific molecules to create remedies for atherosclerosis and restenosis. However, a caveat to this approach is that promoting apoptosis of VSMCs could also lead to the weakening and/or rupture of an already existing atherosclerotic plaque (Kockx and Herman, 2000), but may be a useful approach to treat restenosis following balloon catheterisation and stenting. Interestingly, EGFR transactivation by GPCRs such as AT1R and ET1R are associated with CVD and VSMC proliferation (Forrester et al., 2016), so potentially EGFR transactivation by GPER could potentially contribute to some of the detrimental effects of HRT, although there is no direct evidence for this.

### 4.2 ERs in EC proliferation and monocyte adhesion

Even after the discovery of rapid estrogen signaling, it was thought that EC proliferation and migration is only regulated by genomic E2 signaling and no credit was attributed to rapid signaling. Using disruptive proteins in mouse aorta, the association between ERs and the molecule striatin (vital for rapid signaling) was inhibited together with EC proliferation and migration (Bernelot Moens et al., 2012). However, the disruption of striatin association with ERs was nonselective, therefore this could have also disrupted the interaction between striatin and other proteins involved in the cell cycle. Another study carried out by Lu et al. (2016) on a KRR mutant ERα cell line (triple mutation in receptor that makes it dysfunctional), which confers more specific results than the previous study, also found that rapid signaling of E2 through ERα is indispensable for EC proliferation. In addition, *in vivo* animal studies showed that both ERα and GPER knockout mice experienced higher levels of vascular inflammation after injury and higher levels of monocyte adhesion to ECs, compared to the wildtype mice (Bowling et al., 2014). Furthermore, GPER knockout mice had a significant increase in abdominal fat (Haas et al., 2009), which is consistent with the observation that E2 can lower the circulating levels of LDL (Campos et al., 1997). Knowing that excessive monocyte adhesion and high LDL levels are associated with atherosclerosis and that re-reendothelialization is a crucial step in vascular repair, it is intuitive to deduce that 17β-estradiol is vasculo-protective. However, despite the vasculo-protective properties of E2, the results of HRT studies are inconsistent.

## 5 Hormone replacement therapy studies (HRT)

Since experimental studies have had such success in demonstrating that E2 has vasculo-protective actions, the use of estrogen after menopause, or after ovariectomy, was thought to be essential for preventing or stopping the progression of CVDs, as well as managing menopausal symptoms. However, one of the first, large-scale clinical trials, HERS, showed that conjugated equine estrogens (CEE) plus medroxyprogesterone acetate (MPA) administration had no benefits and, in most cases, it increased the prevalence of coronary heart disease in the first year of treatment, despite the fact that reductions in plasma LDL were recorded (Hulley et al., 1998; Herrington et al., 2000; Viscoli et al., 2001).

CEE is extracted from the urine of pregnant mares and it is a mixture of saturated (such as E1, E3) and unsaturated estrogens (such as equilin) but does not contain E2. Therefore, one of the reasons for the inconsistent results, might be that CEE is less effective than E2. However, this does not explain the increased vascular events seen in HRT studies. Furthermore, MPA and gestodene are progestin medications that can antagonize the protective effects of CEE when administered together. This could explain the deleterious effects of this type of hormone therapy. The WISDOM (Vickers et al., 2007) and PHOREA (Angerer et al., 2001) studies compared the effects of CEE alone, CEE ​+ ​MPA, and CEE ​+ ​gestodene, respectively and discovered that although CEE alone can be beneficial as a primary prevention for CHD because it lowers LDL levels, it increased the risk of breast cancer (Vickers et al., 2007). On the other hand, transdermally administered E2 in combination with statins (a class of drugs that lower cholesterol levels) were proven to have some beneficial effects, which include decreased plasma LDL levels, vasorelaxation and reduced vascular inflammation (Hodis et al., 2001). The ESPRIT study showed no benefit (Cherry et al., 2002), and no benefit was shown in older women with coronary disease treated for 6.8 years with estrogen plus progestin, and in fact increased the rates of venous thromboembolism, biliary tract surgery, and trends in other disease outcomes were also not favorable (Hulley et al., 2002). The EAGAR trial showed slowed atherosclerosis progression in coronary saphenous vein bypass grafts but accelerated disease progression in non-bypassed native coronary arteries (Ouyang et al., 2006), so the effects are mixed and somewhat inconclusive.

Another possible reason for the discrepancy between experimental studies and the clinical trials might be that in most of the studies women had pre-existing CVDs. E2 can cause plaque instability and rupture due to inhibition of VSMC proliferation (see section 4.1) which can lead to myocardial infarction or stroke. Accordingly, the only postmenopausal women in the studies that experienced vasodilation were those that were healthy (Vickers et al., 2007). Moreover, for the same reason, the timing of the therapy (before or after menopause), is critical because as more years pass by, the CVD complications in women become more severe and irreversible. Hypertension, which is caused in part by E2 loss after menopause, also accelerates the progression of atherosclerosis. In the studies summarized in Table 1, women were on average 67 years old, which suggests that they were already postmenopausal for more than 5 years. The ‘critical window hypothesis’ (Maki, 2013) is based on both the observation of these studies and animal studies, which suggested that the protective effects of E2, were more significant if the therapy was initiated before the onset of atherosclerosis. This indicates that estrogen therapy is more likely to be successful when used as primary prevention rather than secondary prevention, since defective arteries are not as responsive to E2 stimulation as healthy arteries are (Maki, 2013).

**Table 1:** Clinical trials summary showing the association of HRT and CVD in postmenopausal women**.**

Trial	Prevention Type	Treatment	Target Group	Benefit/No Benefit	Ref
1998 HERS 1 (4.1 years)	Secondary prevention of CVD	CEE ​+ ​MPA	2763 postmenopausal women >55 with CVD history	No benefit with increased risk of CHD	(Hulley et al., 1998)
2000 ERA (3.2 years)	Secondary prevention of CVD	CEE ​+ ​MPA	309 postmenopausal women >55 with CHD history	No benefit	(Herrington et al., 2000)
2001 WEST (2.8 years)	Secondary prevention of CVD	E2	664 postmenopausal women >55 with history of stroke	No benefit with increased vascular events	(Viscoli et al., 2001)
2001 PHOREA (48 weeks)	Secondary prevention of CVD	E2 and gestodene	321 postmenopausal women >55 with atherosclerosis	No benefit	(Angerer et al., 2001)
2001 EPAT (2 years)	Primary prevention of CVD	E2 ​+ ​statins	199 postmenopausal women with high LDL-C	Slowed atherosclerosis progression if in early menopause	(Hodis et al., 2001)
2002 ESPRIT (2 years)	Secondary prevention of CVD	E2	1017 postmenopausal women 55–69 after an MI	No benefit with increased vaginal bleeding	(Cherry et al., 2002)
2002 HERS 2 (6.8 years)	Secondary prevention of CVD	CEE ​+ ​MPA	2321 postmenopausal women (survivors of 1998 trial)	No benefit with increased risk of ventricular arrythmias	(Hulley et al., 2002)
2006 EAGER (42 months)	Secondary prevention of CVD	Either E2 or E2+MPA	83 postmenopausal women after coronary bypass surgery	No benefit with increased risk of CAD in the remaining healthy coronary vessels	(Ouyang et al., 2006)
2007 WISDOM (10 years)	Primary prevention of CVD	CEE or CEE ​+ ​MPA	5694 healthy postmenopausal women 50-79		(Vickers et al., 2007)
2012 DOPS (16 years)	Primary prevention of CVD	E2 ​+ ​norethisterone	1006 Healthy postmenopausal women 45-58	Significantly reduced risk of mortality HF or MI	(Schierbeck et al., 2012a)
2013 ELITE (5 years)	Primary prevention of CVD and cognitive decline	E2 or E2 ​+ ​progesterone	643 Healthy postmenopausal women 55-65	Slowed atherosclerosis progression if in early menopause	(Hodis et al., 2015)
2019 KEEPS (4 years)	Primary prevention of CVD (carotid intima-media thickness) and coronary calcium	CEE or E2 ​+ ​progesterone	720 Healthy early postmenopausal women 42-58	No benefit. Trend towards reduced coronary calcium and improved bone mineral density	(Miller et al., 2019)

Clinical trials summary showing the association of HRT and CVD in post-menopausal women. CEE: conjugated equine estrogens; MPA: medroxyprogesterone acetate; HERS: Heart and Estrogen/Progestin Replacement Study; ERA: Estrogen Replacement and Atherosclerosis; WEST: Women’s Estrogen for Stroke Trial; PHOREA: Postmenopausal Hormone Replacement Against Atherosclerosis; ESPRIT: Estrogen in the Prevention of Reinfarction Trial; EAGAR: Estrogen and Graft Atherosclerosis Research; WISDOM: Women’s International Study of long Duration Oestrogen after Menopause; EPAT: Estrogen in the Prevention of Atherosclerosis Trial; DOPS: Danish Osteoporosis Prevention Study; ELITE: Early versus Late Intervention Trial with Estradiol; KEEPS: Kronos Early Estrogen Prevention Study.

The issue of the ‘timing hypothesis’ (Hodis et al., 2012) was also addressed by a Danish study, published in 2012, which was a 16 year follow-up of healthy women aged 45–58 who were recently postmenopausal or had perimenopausal symptoms in combination with recorded postmenopausal serum FSH values (Schierbeck et al., 2012a, 2012b). The participants were randomized such that approximately half the women received treatment consisting of triphasic estradiol and norethisterone and women who had undergone hysterectomy (but with recorded FSH values) received 2 ​mg estradiol per day. The control group received no treatment. Intervention was stopped after 11 years due to reports of adverse outcomes in other trials (see Table 1). However, the participants were followed up for an additional 5 years (16 years in total) or achieving the combined end point. The main outcome measure was a composite of death, heart failure and/or myocardial infarction. Interestingly, after 10 years of intervention 16 women in the treatment group experienced the primary endpoint compared to 33 in the control group (p ​= ​0.015). Therefore, the women receiving HRT early after menopause had a significantly reduced risk of mortality, heart failure or MI and without any increased risk of cancer, venous thrombosis or stroke.

It is important to relate these protective effects to possible mechanisms. The authors allude to the fact that, as mentioned above, other studies have shown a beneficial effect of 17-β-estradiol and ethisterone on lipid metabolism, reducing LDL levels, improving endothelial function and reducing carotid intima media thickness (Hulley et al., 1998; Hodis et al., 2001; Effects of estrogen or es, 1995). It is important to again point out that these protective effects are only seen in otherwise healthy women in the absence of overt CVD. It is also reiterated that certain gestogens such as MPA, compared to natural progesterone blunt the positive effects of estrogen. An additional strength of the Danish study is the successful randomization of healthy postmenopausal women and the long-term follow-up, things not achieved in many other trials. Other trials that address the ‘timing hypothesis’ include the ELITE (Hodis et al., 2015) and KRONOS/KEEPS (Miller et al., 2019) studies. Although the findings in these studies with respect to primary and secondary endpoints of CVD and osteoporosis were mixed and somewhat inconclusive, these were only short-term follow-ups (approximately 4 years). It is not the purpose of this review to assess these trials in depth, but rather to focus on possible vascular mechanisms. A systematic review of these clinical trials is covered elsewhere (see for instance (Roberts and Hickey, 2016)).

## 6 Conclusions

**Fig. 5:**
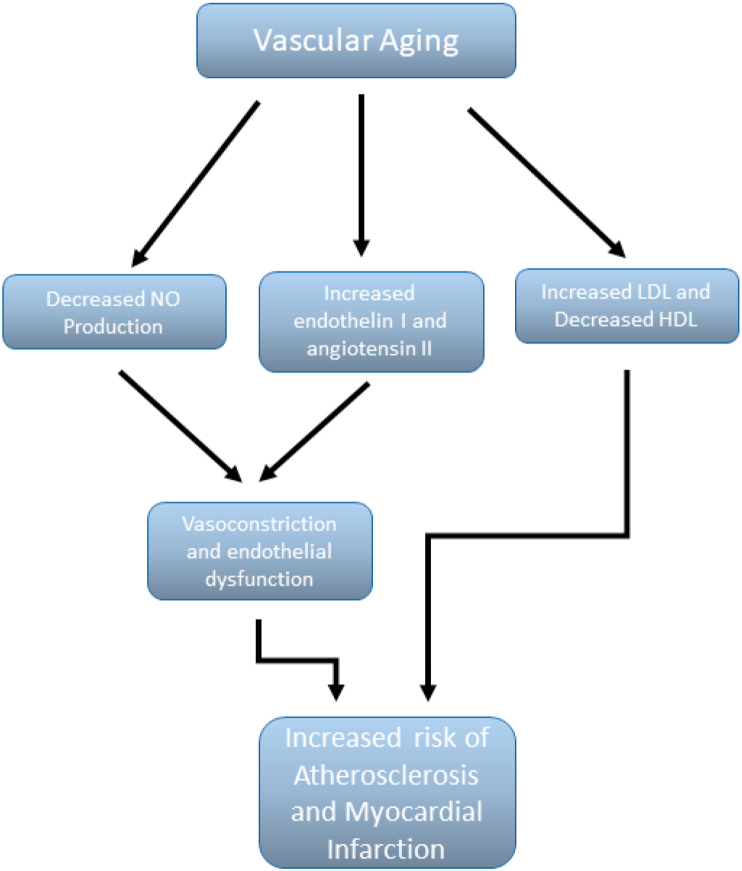
Events linked to vascular aging and E2 loss. MI: myocardial infarction.

Cardiovascular disease is still the number one killer worldwide, being more frequent in post-menopausal women than in pre-menopausal women, which lead to the belief that estradiol (E2) loss, is partly responsible for this. Fig. 5 describes some of the events linked to vascular ageing and E2 loss.

Experimental studies demonstrated that endogenous E2 has vascular protective effects, the best described being that of mediating arterial vasodilation, thus preventing hypertension and the progression of atherosclerosis. By binding to its receptors, E2, activates multiple signaling pathways in both VSMCs and ECs. The PI3K/Akt/eNOS and MAPK/eNOS pathways are activated by the classical receptors ERα and ERβ in endothelial cells and lead to an increased production of NO. Additionally, GPER has been found to mediate most of E2’s rapid signaling mechanisms responsible for VSMC relaxation. This occurs by activating BKca channels and initiating the cAMP/PKA/RhoA pathway. However, the results of the studies varied within different vascular beds, with GPER mediating vasoconstriction in rat kidney arteries via the transactivation of EGFR and ERK1/2. Rapid E2 signaling is also necessary for VSMC apoptosis, EC proliferation, inhibition of monocyte adhesion and decreasing LDL levels, all mechanisms that are vital for vascular healing and preventing atherosclerosis. Furthermore, estrogen is also involved in genomic signaling (e.g., via MAPK-mitogen activated protein kinase and direct nuclearlocalisation/genomic effects), which was not discussed in detail in this review, but also plays an important role in its protective effects and has been covered elsewhere (Murphy, 2011; Menazza and Murphy, 2016).

Studying ER signaling mechanisms and subsequently discovering therapies to treat these complications or at least prevent them is harder than originally thought, not least because most antagonists for either ERα or ERβ are agonists for GPER. Although HRT studies showed increased risk of CV events, they also opened new doors for further research, since it was observed that these results are influenced by different factors such as: type of hormone treatment; timing and duration of HRT administration; patient’s health, estrogen receptors levels; their sensitivity to E2 and the changes in the structure of the vascular wall.

In conclusion, in order to fully exploit E2 protective actions and get better clinical trial results, future research should be more focused on characterizing the receptors’ function and distribution in different cellular and tissue compartments, for instance in both arteries and veins, and the molecules and pathways that E2 itself, and ERs interact with. For instance, the interaction between GPER1 and ERα36 in immune cells is a good case in point (Notas et al., 2020). This could lead to the discovery of new signaling pathways that are responsible for the deleterious effects observed in HRT, which would make their specific inhibition possible. So could E2 be deleterious in post-menopausal women based on our current understanding of the signaling described in this review ? For instance, as we mentioned above GPER can induce EGFR transactivation which, in general, contributes to CVD and it is possible that GPER could be more pro-contractile in post-menopausal women in addition to the fact that EC dysfunction impairs the beneficial actions of E2 via ERα and ERβ in general. So, the underlying health of the vasculature seems to be critical in determining whether E2 effects in an HRT context are beneficial or detrimental, since this probably determines whether the classical ERs, ERα and ERβ in particular, are protective or deleterious due to pro- or anti-oxidant effects in the vasculature. Of particular importance is the role of GPER1 in immune cells, particularly in the antagonism of TLR4-dependent pro-inflammatory pathways (see (Notas et al., 2020)), which also highlights, perhaps, a central role of NFκB-dependent signaling in pro-inflammatory vascular disease and atherosclerosis progression in particular. This could be relevant given that the relative and regional expression of the three receptors varies with age and with hormonal status.

Another question is whether or not menopausal EC dysfunction is really irreversible. If E2 and PG alter expression of their own receptors, there may be negative and/or positive feedback. One problem is that menopause is almost only seen in humans, so longitudinal studies in animal models are difficult to achieve. Nevertheless, the protective role of E2 in general is not disputed, certainly from experimental studies. The apparent failure of HRT trials in humans compared to the largely positive results of experimental studies is almost certainly due to the presence of vascular disease and dysfunction which alters the response to E2; alterations in the distribution and expression of different ERs; and in differences in the response of different ERs to different agonists, ie, natural versus synthetic estrogens. Perhaps in the future there needs to be more focus on the role of specific ERs, the downstream signaling pathways that they interact with and their agonists. The development of agonists for specific receptors, in particular GPER, for instance maybe one way forward. Also, modulation of E2 bioavailability and receptor expression/activity may be effective approaches, for instance by modulating aromatase activity. Interestingly this approach may be useful in men as well as post-menopausal women, so there is hope for the future.

## Credit Author statement

Ana-Roberta Nita: provided the first draft, made corrections to subsequent drafts

Greg A. Knock: Edited and provided comments on the first and second drafts

Richard J. Heads: Edited the first and second drafts, made extensive changes, prepared the final draft for submission, did all the formatting, prepared all the diagrams, did all the revision to the final revised submission.

## Declaration of competing interest

The authors declare that they have no known competing financial interests or personal relationships that could have appeared to influence the work reported in this paper.
